# Rhegmatogenous Retinal Detachment with Spontaneous Dialysis of the Ora Serrata in Neurofibromatosis Type 1: A Case Report

**DOI:** 10.31729/jnma.7392

**Published:** 2022-06-30

**Authors:** Richa Makaju Shrestha, Swechha Bhatt, Pooja Shrestha, Prakash Sapkota, Rajani Keshari, Anu Manandhar, Iwa Bhattarai

**Affiliations:** 1 Department of Ophthalmology, Kathmandu University School of Medical Sciences, Dhulikhel Hospital, Dhulikhel, Kavre, Nepal; 2 Kathmandu University School of Medical Sciences, Dhulikhel Hospital, Dhulikhel, Kavre, Nepal; 3 Department of Internal Medicine, Kathmandu University School of Medical Sciences, Dhulikhel Hospital, Dhulikhel, Kavre, Nepal

**Keywords:** *case reports*, *neurofibromatosis type 1*, *ora serrata*, *retinal*, *retinal detachment*

## Abstract

Neurofibromatosis type 1 is a genetic disorder that follows an autosomal dominant pattern of inheritance. Ocular involvement is not uncommon, but spontaneous dialysis of the retina in the absence of a history of trauma is a rare clinical entity. Rare cases of retinal involvement such as retinal detachment or dialysis of ora serrata could be linked with the abnormal cell-matrix formation in neurofibromatosis type 1. Here, we present a case of a 36-year-old man having Neurofibromatosis Type 1 with spontaneous dialysis of ora serrata without prior history of ocular trauma. A routine fundoscopic examination should be done in addition to the examination of the anterior chamber in patients with neurofibromatosis type 1 despite the absence of ocular complaints.

## INTRODUCTION

Neurofibromatosis Type 1 (NF 1) (Von recklinghausen's disease) is an autosomal-dominant genetic disorder due to a loss of function mutation in the NF 1 gene,^1^ located on chromosome 17q11.2.^2^ The patient presents with neurocutaneous manifestations like cafe-au-lait macules, axillary and/or inguinal freckling, and neurofibromas.^1^ Ophthalmic manifestation of NF1 includes Lisch nodules, optic nerve gliomas, plexiform neurofibromas, retinal astrocytic hamartomas, and choroidal nodules.^3^ Amongst these, retinal lesions and choroidal lesions are less common.^3-5^ We report an unusual case of rhegmatogenous retinal detachment associated with spontaneous dialysis of the ora serrata in the left eye in a young male with neurofibromatosis type 1, with no history of ocular trauma and tumor.

## CASE REPORT

**Figure 1:**
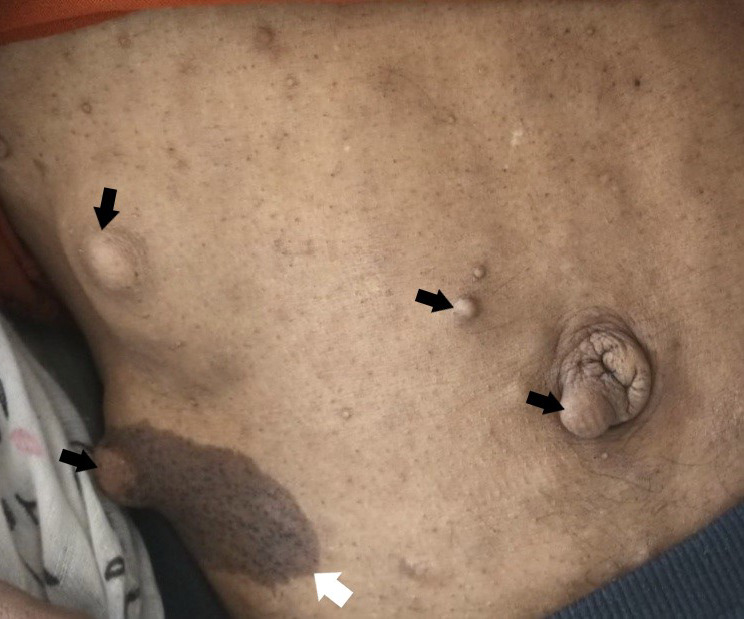
Multiple neurofibromas (black arrows) with cafe au lait spot (white arrow) indicative of neurofibromatosis type 1.

A 36-year-old male presented with chief complaints of Shortness of Breath (SOB) and cough for 2 months. SOB was gradually progressive and of modified Medical Research Council (mMRC) grade 3 at the time of presentation. It was relieved while lying on the left side but aggravated on lying on the right side. The cough was productive, whitish, and non-blood mixed. He also had a history of night sweats and weight loss. On examination, 7-9 dark brown coloured cafe au lait spots of approximately 3x2 cm size each were present along with multiple neurofibromas over the trunk (Figure 1).

**Figure 2:**
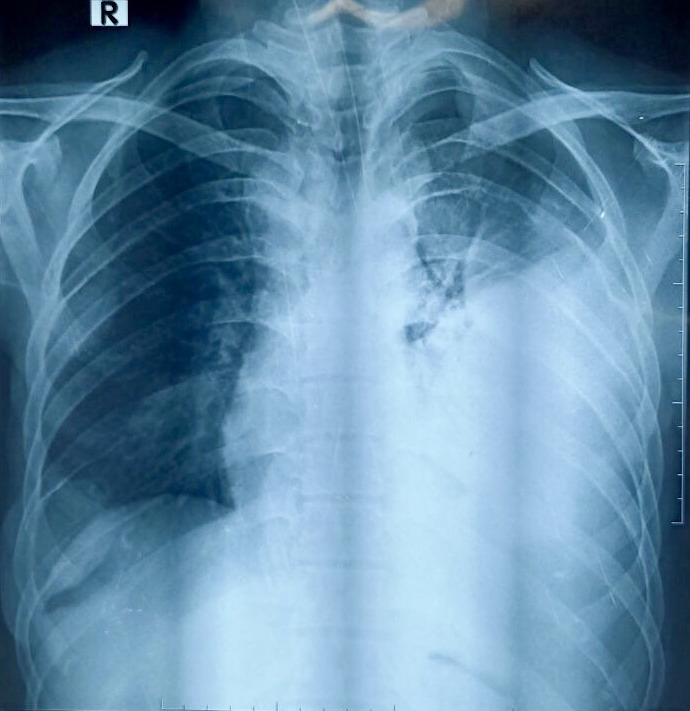
CXR showing left-sided pleural effusion.

There were several non-tender cervical lymph nodes palpated of which the largest lymph node measured approximately 20 mm. Following clinical examination, a Chest X-ray (CXR) was done which showed obliteration of left costophrenic and cardiophrenic angles with a homogenous opacity in the left middle and lower lung zones without tracheal or mediastinal shift, suggestive of left-sided pleural effusion (Figure 2).

To confirm the diagnosis, a Contrast Enhanced Computed Tomography (CECT) chest was done which showed left-sided moderate pleural effusion with normal lung parenchyma along with features suggestive of plexiform neurofibromatosis. Pleural fluid analysis was done which showed lymphocytic effusion with high pleural fluid adenosine deaminase (55 IU/l). The patient was treated with anti-tubercular treatment with a diagnosis of neurofibromatosis with left-sided tubercular pleural effusion. He had no significant family history related to neurofibromatosis or tuberculosis. He was referred to the Department of Ophthalmology for ocular evaluation for NF 1.

On further inquiry in the Department of Ophthalmology, he gave no history of ocular symptoms. There was no history of trauma to the eye and preceding ocular surgeries. On ophthalmological evaluation, the patient had a best-corrected visual acuity of 6/9 on the right eye Oculus Dexter (OD), and 1/60 on the left eye Oculus Sinister (OS) with no error noted. Further evaluation showed left exotropia of an eight-degree prism dioptre. Pupillary examination showed no anisocoria; however, a positive Relative Afferent Pupillary Defect (RAPD) of grade 2 was present on the left eye (OS). There was no evidence of angle recession, iridodialysis, phacodonesis, or choroidal ruptures. Slit-lamp examination revealed no iris Lisch nodules in either eye.

**Figure 3:**
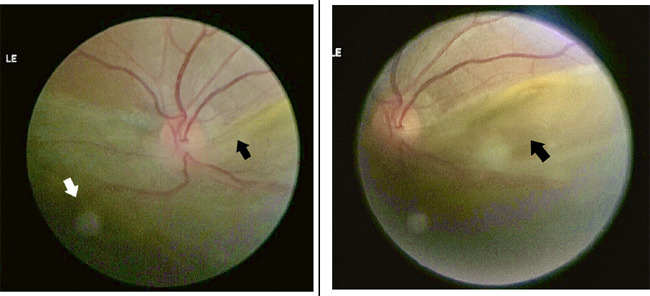
Posterior segment photograph (taken by fundus camera) of the left eye showing inferior retinal detachment (black arrow) involving the macula and dialysis of ora serrata (white arrow).

Fundoscopy following pupil dilation revealed inferiorretinal detachment with the macula off in the left eye. The right eye was normal. The dialysis of the ora serrata was noted from the 4 o'clock to the 7 o'clock positions (Figure 3).

**Figure 4:**
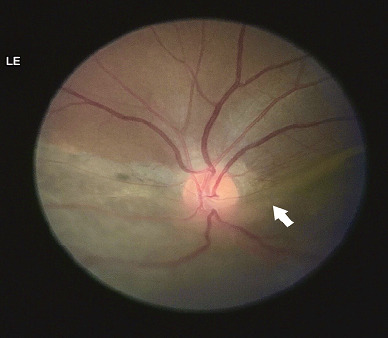
Follow-up fundus findings of the left eye with persistent inferior retinal detachment (white arrow).

**Figure 5:**
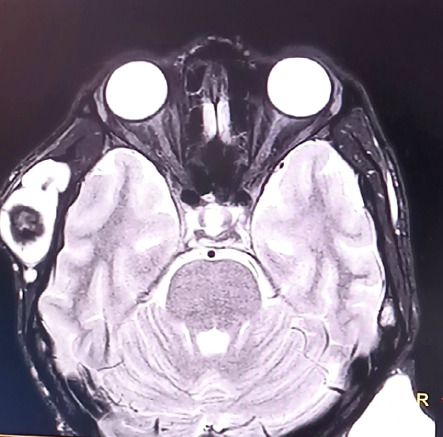
An axial T2-weighted MRI of the brain shows both the orbits with no features of surface occupying lesions.

The detailed ocular examination did not yield clinical evidence suggesting ocular trauma, and the patient repeatedly denied the same. Rhegmatogenous retinal detachment with spontaneous dialysis of the ora serrata in the left eye was diagnosed. On follow-up after 3 weeks, he was admitted with a lung abscess, for which chest tube insertion and drainage of the fluid were done. However, he had no ocular complaints, and the ophthalmic evaluation revealed no deterioration of vision. Even so, the ocular findings of Rhegmatogenous Retinal Detachment (RRD) with spontaneous retinal dialysis persisted (Figure 4), raising suspicion of an underlying tumor. Following this, an Magnetic Resonance Imaging (MRI) of the brain and orbit showed no orbital or cranial pathologies (Figure 5).

## DISCUSSION

Neurofibromatosis type 1 (NF 1) is characterized by abnormal growths of epithelial, mesothelial, and endothelial components.^6^ The cutaneous features include cafe-au-lait macules, axillary and groin freckling (Crowe sign), dermal tumors, etc.^1^ It can also affect the gastrointestinal tract, eye, musculoskeletal and neurovascular systems.^1^ The ocular manifestations can range from benign growths like iris hamartoma (Lisch nodules) and neurofibromas to malignant tumors like Optic Pathway Glioma (OPG).^1-3^

Retinal detachment in patients with NF 1 usually occurs following mechanical trauma to the eye or with retinal tumors like hamartomas and capillary hemangioma.^7^ Likewise, retinal dialysis also occurs secondary to trauma; however, a few inherited spontaneous retinal dialysis cases have also been reported.^8^ Retinal detachment with spontaneous dialysis of ora serrata in the absence of a history of ocular injury or tumor has rarely been reported in patients with neurofibromatosis type 1.^3^

The vitreous base contains numerous fibroblasts in its cortex, which form collagen fibrils that insert through the basal lamina of the pars plana and peripheral retina.^3^ Fibroblasts migrate and proliferate in this region following vitreous haemorrhage and penetrating ocular trauma and are thought to be responsible for the vitreous base avulsion.^9^ In NF 1, vitreoretinal pathologies are linked with abnormal cellular proliferation in the vitreous base even in the absence of previous ocular pathology.^6,10^ One article hypothesized that there is Matrix Metalloproteinase 1 (MMP1) downregulation in stromal cells in NF 1 leading to disturbance in the collagenolytic process and thereby accumulation of abnormal fibroblast and neo-collagen in the ocular region in NF 1.^10^ Another study showed that there is decreased receptor binding of Epidermal Growth Factor (EGF) in NF 1, which might lead to membrane defect in the retina.^6^ These factors alter the cellular matrix of the vitreous base resulting in reduced production of collagen fibers that keep the peripheral neurosensory retina intact.^6,9,10^

In a pediatric case study, the ocular examination revealed retinal detachment with dialysis of the ora serrata in the left eye, similar to our case, the patient had no ocular symptoms and no history of ocular trauma.^3^ Unlike in their case, our patient had diminished visual acuity in the left eye noted after examination along with exotropia and grade 2 RAPD therefore, we performed an MRI of the brain instead of an exploration of the retina to rule out tumors. In their case, the underlying defect was corrected by ophthalmological intervention but this approach was not done in our case since the patient had asymptomatic chronic RRD. We opted to observe the patient with routine follow-up as the risk of progression of the RRD outweighed the benefit of surgical repair.

Although retinal dialysis is a rare entity in cases with NF 1, with this case, we would like to emphasize performing routine funduscopic examination under mydriatics in all the patients with NF 1 even in the absence of ocular complaints in addition to evaluating the anterior segment. Furthermore, imaging modalities like MRI of the brain should be employed to rule out underlying lesions. This will not only aid in ruling out the presence of ocular lesions but also help in diagnosing any obscured asymptomatic retinal pathologies like spontaneous retinal dialysis, as in our case thereby, preventing vision-threatening complications.
